# Medical internship training during the COVID-19 pandemic – A case of ‘sacrificial pawns’ or not?

**DOI:** 10.4102/phcfm.v14i1.3103

**Published:** 2022-01-13

**Authors:** Veena S. Singaram, Kimesh Naidoo, Labby Ramrathan

**Affiliations:** 1 Department of Clinical and Professional Practice, School of Clinical Medicine, College of Health Sciences, University of KwaZulu-Natal, Durban, South Africa; 2 Department of Paediatrics and Child Health, School of Clinical Medicine, College of Health Sciences, University of KwaZulu-Natal, Durban, South Africa; 3 Department of Teacher Development Studies, Faculty of Education, University of KwaZulu Natal, Durban, South Africa

**Keywords:** medical interns, stress, burnout, well-being, training, workplace, competencies, self-regulated learning

## Abstract

**Background:**

Newly qualified medical practitioners in South Africa (SA) are part of the frontline health care workers who face Africa’s most severe coronavirus disease 2019 (COVID-19) pandemic. The experiences of interns during the pandemic reflect SA’s preparedness to respond in a crisis and inform strategies that could be adopted to balance training and service in resource-challenged contexts.

**Aim:**

To explore the strengths, weaknesses, opportunities and threats posed during the first wave of the COVID-19 pandemic as reflected on by interns within the clinical training platforms in SA.

**Setting:**

Public hospitals in KwaZulu-Natal.

**Methods:**

An online questionnaire consisting of eight open-ended questions based on the SWOT framework related to personal and professional perspectives to clinical training during the COVID-19 pandemic was developed using SurveyMonkey. All data were collected remotely via social media platforms. Data were thematically analysed.

**Results:**

Forty-six interns reflected on personal and systemic challenges as the major threats and weaknesses in intern training during the COVID-19 pandemic. Extrapolating on strengths and opportunities, there were three overarching learnings interns reflected on. These related to being a medical professional, communities of practice and the development and enhancement of clinical and non-clinical competencies. Existing challenges in the environment exacerbated the threats posed by COVID-19 and innovative strategies related to improving support, feedback, broadening the intern curriculum and online training.

**Conclusion:**

Although the clinical environment where interns learn and work is often stressful and overpowered by high service burdens, there are unique opportunities to enhance self-directed learning and graduate competencies, even in the midst of the COVID-19 pandemic.

## Introduction

Coronavirus disease 2019 (COVID-19) has substantially disrupted conventional medical practice and training, as this sector became the focal point of heightened engagement because of their crucial role in managing this evolving pandemic. Within this context, a focus on medical interns as part of the frontline workers was needed in order to understand and put in place strategies to support them as emerging medical practitioners. Hence, the central question that guided this article was as follows: ‘What learning opportunities were possible for medical interns within the context of the COVID-19 pandemic in South Africa?’

Medical interns are at a formative stage of their careers and require a focus on both service delivery and training. For this next generation of health care workers who will lead the response to forthcoming pandemics, it is vital to ensure that training is optimally managed and that interns learn about ‘leadership, teamwork, and crisis management’.^1^ These professional characteristics are included within a comprehensive Intern training curriculum regulated by the Health Professions Council of South Africa (HPCSA). This curriculum includes specific and general clinical and professional learning requirements elucidated in a logbook that requires completion by all interns prior to exit.

Furthermore, it is at these times of crisis that physician educators are known to model professionalism to their trainees.^2^ The COVID-19 pandemic created new expectations for both senior and emerging medical practitioners to ensure that all staff are well informed and prepared to remain on the frontlines whilst education continues.^2^ This expectation includes that senior staff stand ‘shoulder to shoulder’ with emerging staff to deliver care collaboratively.^2^

South Africa (SA), a resource-constrained setting, is the African country most severely affected by COVID-19.^3^ The country’s public health system, already burdened with multiple epidemics of , tuberculosis and trauma, has had to prepare its health care workers for the COVID-19 pandemic.^3,4^ This threat, also faced by overburdened public health systems across Africa, was recognised early, and calls were made for a comprehensive strategy to prepare and respond with capacity building amongst health care workers.^5^ Despite such efforts, the effects of the COVID-19 pandemic have been stark, with significant numbers of frontline health care workers being infected or demising.^6^ The group of frontline health care workers in SA includes medical interns. In 2020, 2369 interns were appointed in the largest deployment in South African history.^7^ The SA interns work long hours in resource-challenged hospitals where supervision is not uniformly optimal.^8,9,10^ High levels of stress, anxiety and burnout have been documented amongst interns in SA prior to the COVID-19 pandemic.^11^

In this study, we aim to explore the strengths, weaknesses, opportunities and threats (SWOT) of medical internship clinical training within the context of the COVID-19 pandemic. After performing this SWOT analysis, we argue that, although interns learning and working are often stressed or overwhelmed by high service burdens, especially in resource-challenged settings, there are unique opportunities to enhance self-directed learning and graduate competencies that are enhanced by communities of practice at worksites.^12^

## Methods

### Study design

This was a descriptive exploratory qualitative study conducted using a SWOT framework. A SWOT framework is used as an effective situation analysis technique that entails identifying the strengths, weaknesses, threats and opportunities that one encounters in a system.^13^ It is also used for both determining the resource capabilities of a system and as a results-orientated strategic planning tool.^14^ The COVID-19 pandemic provided an unusual context within which interns found themselves and as such their intended learnings may have been disrupted. The exploratory qualitative study provided the scope to explore what learning was possible, what was not, what was unintended and what was opportunistic within the COVID-19 context.

### Setting

Medical internship in SA incorporates a 24-month salaried training period that certifies medical practitioners for independent practice in the country. Interns have a 3-month rotation through each of the major disciplines, that is, internal medicine, general surgery, obstetrics and gynaecology, paediatrics and family medicine. They have shorter rotations of two months each through anaesthesia, orthopaedics and psychiatry. Interns work in the frontlines of public sector hospitals serving largely non-fee-paying patients from the lower income quintiles.^14^ Interns in this study were from public hospitals across KwaZulu-Natal’s largest cities and towns. These hospitals are governed by the Department of Health in each province under the umbrella of the National Department of Health (NDOH).^15^ The internship programme is managed by the NDOH through a professional accrediting body, the HPCSA Internship Subcommittee.^15^ Higher education bodies do not directly oversee internship, although many of the intern supervisors are affiliated to various medical schools.^15^

### Sample

Medical interns were invited by the investigators using snowball sampling as it was challenging to access respondents with the target characteristics.^16^ The target characteristics for purposive sampling was first- and second-year interns working within the public sector hospitals in KZN. The choice of snowball sampling was informed by COVID-19 contexts that compromised the process of determining the exact population from which to extract the sample. Data saturation was established when no new categories of responses or recurring responses were received by the subjects. Initially six medical intern representatives based at different hospitals in KZN were contacted to participate in the study and share the questionnaire link with other interns. At a sample size of 46, it became apparent that no new responses were being received and at this point no further request for referral was made. Hence, the snowball sampling was most appropriate for the intent of this study within the COVID-19 context.

### Data collection

An online questionnaire with eight open-ended questions was developed by the primary investigator and collaborators based on the SWOT framework focusing on the personal and professional SWOT related to clinical training during the first wave of the COVID-19 pandemic. A biographical (e.g. age, gender and year of internship) tick box section was included. The questionnaire was developed on SurveyMonkey (SurveyMonkey Inc., San Mateo, California, United States [US], www.surveymonkey.com) and made available to the sampled interns via the online hyperlink. The link was sent via the social media platform, WhatsApp. The questionnaire was completed anonymously.

### Data analysis

An *apriori*, deductive process using the SWOT framework was used in the analytical process. The SWOT framework formed the basis for the *apriori* categorising of the data for analysis. This process of analysis allowed for the largely descriptive aspect of the study. The data were read several times to gain familiarity with the responses received from the respondents. The responses in each of these categories were then framed by respondents’ understanding of their personal and professional responses in each of these categories to clinical training during the pandemic. The responses in each were then collated across all interns in each category and then thematically analysed. Hence the primary analytical method used was thematic content analysis.^17^ Thematic analysis involves looking across the data set to identify common issues that recur and identifying the main themes that summarise all the views collected.^18^ The authors V.S.S. and K.N. individually interpreted and immersed themselves in the data by reading and rereading it several times to identify codes and emergent themes.^19^

To enhance the trustworthiness of the authors’ interpretations, a research assistant was employed to codify and categorise the data. Themes and subthemes were identified, compared and discussed by V.S.S., K.N. and L.R. until consensus was reached. The initial six interns were representatives elected by the cohort of interns based at the representative hospital complex. There were no prior relationships with these interns and the researchers. Because of the anonymity of the questionnaire, the other respondents were unknown to the researchers.

V.S.S. is a medical educationalist involved in both clinical training and academic management. Her role focused on the intended learning from internship programmes to explore what was possible, what was not possible and what lessons can be learnt during a pandemic to inform curriculum issues. K.N. is a clinician and clinical intern trainer who works within COVID units. His interest was on preparing students for internship and insights from this study opened up opportunities to review training programmes for future interns. L.R. is an educationalist involved in scholarship that includes health professional education. Exploring teaching and learning opportunities, especially within constrained and disruptive contexts provides L.R. the scope to contribute to the scholarship of teaching and learning.

A summary of the themes, with supporting quotes, is given in the following section. To keep their identities anonymous, the participants (P) are represented by numbers, which are included with the quotes for purposes of trustworthiness and confirmability.

### Ethical considerations

This study received approval from the University of KwaZulu-Natal Ethics Committee (HSSREC/00001306/2020). All participants provided informed consent. All methods were carried out in accordance with relevant guidelines and regulations.

## Results

**FIGURE 1:**
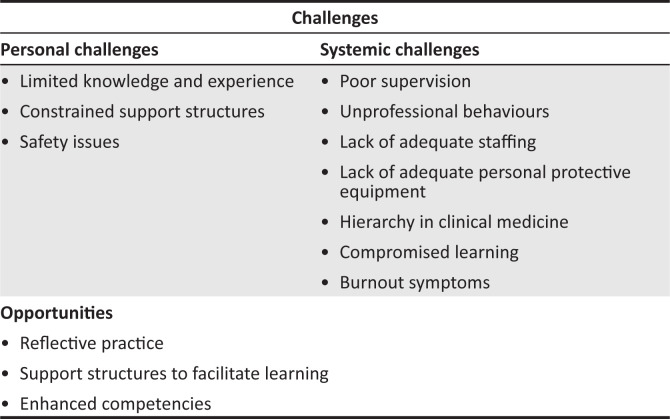
Major themes and subthemes.

Of the sampled group of 46 interns, 42% were in the first year of their 2-year internship period, 56% in the second year and 2% unknown. The majority of the interns were female (56%) and single (66%), and most did not have children (91%). Of the total number of 226 responses to open-ended items, 40% related to strengths, highlighting positive aspects, whilst 46% highlighted the negative aspects related to weaknesses (20%) and threats (26%). Fifteen per cent described opportunities that arose as a result of the COVID-19 pandemic. The major themes and subthemes are illustrated in Figure 1.

### Personal challenges

Interns, having recently graduated from medical school, were thrust into the frontlines during the COVID-19 pandemic. Three themes that illustrated their feelings of personal limitations, vulnerability and neglect were identified.

#### Limited knowledge and experience

The subtheme of inexperience was unsurprisingly reported by the interns. This often came in the form of phrases that reflected a lack of knowledge or insufficient knowledge:

‘Limited knowledge to function independently.’ (P11, male, 1st year)

‘I still require experience in the rotations.’ (P24, female, 2nd year)

#### Constrained support structures

The lockdown restrictions and poor access to support structures contributed to or exacerbated stress and anxiety:

‘Stress caused by lack of access to support structures [*family and friends*] and lack of stress release – socialising, being outdoors, going on trips.’ (P36, female, 2nd year)

#### Safety issues

A major subtheme identified from the data and viewed as a threat was the safety concerns many interns expressed about being infected by COVID-19. These concerns extended to their significant others such as family, partners and older relatives with whom they reside:

‘How are we supposed to get all the training we need when we are continuously praying we don’t die or go home and spread the disease?’ (P27, male, 2nd year)

‘Living with elderly family members and the need to not want to place them at risk, that is, move out of the house during COVID [*coronavirus disease*] time.’ (P1, male, 2nd year)

### Systemic challenges

Systemic challenges related to existing fault lines within the public health system, safety issues specific to COVID-19, compromised learning and the exacerbation of burnout symptoms amongst already anxious and overburdened interns.

#### Poor supervision

The subthemes around supervision tended to reflect negatively on supervisors. The interns reported weaknesses in their positions because of what they felt was attributed to their status as interns. They felt that supervisors neglected interacting with them regularly on important issues such as feedback and debriefings:

‘Meetings and feedback don’t happen anymore.’ (P21, female, 2nd year)

‘High-stress environment, mental health “testing,” limited time and space for debriefing, lack of directed information.’ (P18, female, 2nd year)

#### Unprofessional behaviours

The interns observed unprofessional behaviours in their supervisors and senior medical staff, which also hindered their learning and increased their fears:

‘Senior medical staff have terrible bedside manner, which is now exaggerated with the pandemic.’ (P10, male, 2nd year)

‘The ever-present threat of being dismissed if one refused to go to the COVID [*coronavirus disease*] unit to be abused hinders one’s willingness to learn quite substantially.’ (P27, male, 2nd year)

#### Lack of adequate staffing

The lack of adequate staffing was seen to be exacerbated by redeployment to new COVID-19 related services areas, new scheduling, staff being quarantined and absenteeism:

‘Staff shortages means more calls.’ (P10, female, 2nd year)

‘Other doctors not showing up for work because they are scared to get infected, leaving the rest of us understaffed and work overloaded.’ (P26, male, 2nd year)

#### Lack of adequate personal protective equipment

Many interns expressed views related to poor access to the most optimal types of personal protective equipment (PPE) required as well as concerns with the provision of adequate quantity and quality of PPE. The interns therefore had to make do with what was at their disposal, even if it means reneging on their own personal safety precautions. The clinical setting in which the interns work then comes to be experienced as a setting of compromise, which is often accompanied by feelings of fear.

‘Unavailability of proper PPE [*personal protective equipment*] – lately our antiseptic hand rub is of poor quality.’ (P13, female, 2nd year)

‘Not enough PPEs [*personal protective equipment*] and often had to re-use masks from the previous day or worse.’ (P31, male, 2nd year)

#### Hierarchy in clinical medicine

A major subtheme to emerge from the data related to what interns viewed as exacerbated negative hierarchical relationships already present in clinical medicine. The interns had very strong words for the hierarchical structure of power in the medical settings in which they worked:

‘[*We are*] used as sacrificial pawns … 100% clear that our lives mean less.’ (P27, male, 2nd year)

‘Dissatisfaction with a system which abuses the lowest rank, using us as cannon fodder instead of sending senior clinicians first.’ (P45, male, 1st year)

#### Compromised learning

The interns expressed concerns over the decreased exposure to adequate patient numbers, pathology and training, as many patients defaulted on scheduled hospital visits. In addition, time away from work because of quarantine or infection and decreased direct patient physical contact compromised learning.

‘Less patients, less procedures, no training programmes, no academic classes.’ (P7, female, 2nd year)

‘Tutorials have largely been cancelled … meetings and feedback don’t happen anymore.’ (P21, female, 2nd year)

#### Burnout symptoms

The interns indicated that fear, anxiety, stress and being overwhelmed were emotional responses they were experiencing within themselves as a result of the COVID-19 pandemic. These emotions were seen to aggravate an existing stressful internship known to have high levels of burnout.

‘It’s overwhelming and scary, almost every patient is now becoming a PUI [*person under investigation*]and we don’t know if we are adequately protected.’ (P19, male, 1st year)

‘Internship is already so terrifying and emotionally abusive … interns are depressed and emotionally affected, but because we are just “interns”, we are gleefully seen by higher-ranking members of the health system as “they are fine” and “it makes us stronger.”’ (P27, male, 2nd year)

### Opportunities

Three major opportunities were identified by the interns. The first related to reflective practice and the second to support structures that facilitated learning. The third related to the development and enhancement of clinical and non-clinical competencies.

#### Reflective practice

The interns gained insights of self and practice as they reflected on their strengths as resources to leverage in order to sustain clinical work and training as they experienced the pandemic. Being able to work under pressure and manage change were identified as key skills to cope amidst the changes experienced. These included the innate capacity of the interns’ resilience-building skills as expressed by themselves as being ‘self-motivated’ (P15, male, 1st year); showing ‘adaptability’ (P30, female, 1st year); ‘flexibility’ (P33, female, 1st year) and ‘optimism’ (P40, female, 1st year).

In addition, the interns expressed insights regarding the development of their professional behaviours as being ‘independent’ (P12, male, 2nd year); ‘being responsible’ (P16, female, 1st year); showing ‘persistence’ (P29, male, 1st year), ‘discipline’ (P40, female, 1st year) and the ‘development of [*the*] ability to work in many different work environments’ (P30, mlae, 2nd year) as advantageous during the COVID 19 pandemic.

To combat the stresses of the uncertainty associated with the evolving pandemic, they reflected on their positive attitudes which also included behaviours of being a ‘fast worker’ (P24, female, 2nd year) and:

‘I’m just an all-around badass taking it all in my stride.’ (P6, male, 2nd year)

‘I am always around looking for ways to get involved.’ (P29, male, 1st year)

### Coping skills

The interns identified strategies when working in teams that enabled them to constructively contribute meaningfully to outputs with the constantly changing demands:

‘Level-headedness with regard to having a logical rather than emotional response to persistent changes.’ (P30, male, 2nd year)

‘Crisis management, organisation, taking leadership roles, compassion within team settings and being a team player.’ (P33, female, 1st year)

#### Support structures to facilitate learning

The interns identified their colleagues, friends and family as positive support systems during the COVID-19 pandemic. In a few instances, they described their superiors as sources of access to support. Often, they collaborated with each other and worked in teams in order to provide understanding to each other, learn together and teach each other. Online Zoom calls were a popular source of support in terms of bridging the knowledge gaps that interns felt they possessed regarding COVID-19:

‘Fellow intern support … can teach each other and have support.’ (P17, male, 2nd year)

‘Working in a team, having superiors available to consult whenever necessary, training via online Zoom calls.’ (P24, female, 2nd year)

#### Enhanced competencies

New learning opportunities in clinical skills specific to the COVID-19 pandemic and the management of patients in high-care situations were highlighted by the interns.

‘How to treat hypoxic pneumonias.’ (P14, male, 2nd year)

‘Emphasis on improving intubation skills.’ (P36, male, 2nd year)

Many interns valued the knowledge and training of infection prevention and control (IPC) practices as strengths in being ready to deal with the pandemic as frontline workers.

‘Knowing the value of IPC [*infection prevention and control*], pandemic preparedness and a first-person perspective of a disaster.’ (P12, male, 2nd year)

‘We have learnt to practice strict sanitising methods. In other words, how to continue working in a hospital despite a pandemic.’ (P25, female, 1st year)

The interns often reported using whatever was at their disposal and developing on-the-go knowledge in order to deal with what confronted them in their daily routines of clinical care. It is encouraging to note the collaborative and leadership development of the interns as they indicated their acceptance of learning with their seniors as opposed to the usual expectation of learning from their seniors:

‘We are learning with our seniors about this virus as we go along every day in the hospital.’ (P29, male, 1st year)

Even though the interns described themselves as relatively structurally powerless, it was interesting to see some reports of health advocacy having emerged out of this situation. Health advocacy often took the form of spreading the correct knowledge about COVID-19 to colleagues and friends, and discarding propaganda. It also developed in cases where the interns took active steps to implement protocols despite resource constraints in their departments:

‘Health advocacy and education in terms of social media … and propaganda.’ (P5, male, 2nd year)

‘Development of health advocates in each department.’ (P12, male, 2nd year)

## Discussion

The discussion unfolds within three key areas and is guided by the themes and subthemes outlined in Figure 1.

### On becoming a medical doctor during a global medical crisis

In the time of a disease crisis, the professional identity construction seems to receive some attention. Professional identity has been conceptualised as the adoption and development of identifiable characteristics specific to that profession.^20^ The subjects in this study alluded to this professional identity construction. They indicated that they began to develop a sense of intrigue about the disease, worked more efficiently in a high-demand environment, and developed an attitude of getting involved and working across different disciplines. All of this relates to the attitudinal and soft skills that are value-based, and core to being a professional. There are, however, some threats to this professional identity construction. These include the comments: ‘[*being*] abused hinders one’s willingness to learn quite substantially’ (P27, male, 2nd year); ‘lack of stress release – socialising, being outdoors, going on trips’ (P36, male, 2nd year); and ‘being dismissed if one refused to go to the COVID unit’ (P27, male, 2nd year). Under appropriate management, these threats can be addressed and curbed to a point where they do not have a major influence on the professional identity construction process.

## From hierarchical learning to communities of practice

The data suggest that hierarchical learning from experts to interns is replaced with collaborative learning processes with the emphasis on learning rather than teaching in times of unprecedented crisis conditions. Wenger’s concept of communities of practice brings together a significant number of individuals, which amongst others include differing expertise, experience, knowledge and positionality, within a learning space where the focus is on learning.^21^ Each individual within this community of practice shares in the learning process, drawing from individual strengths and supporting others in their learning endeavours. In the context of the pandemic, communities of practice formed the basis of the teaching and learning community wherein supervisors, interns and others could engage meaningfully to respond medically to the COVID-19 pandemic, being the centre of engagement. This community of practice mode of learning is not immune to weaknesses and threats. The most common weaknesses, as expressed by the subjects, include a reduction in feedback and debriefing sessions on intended learning. In this instance, the planned learning is replaced by on-the-job learning and, as such, what was planned for learning during a normal internship period is marginalised by the prevailing medical emergency. Hence, emergency learning takes centre stage.

### Development and enhancement of clinical and non-clinical competencies within the context of a global medical pandemic

Aoki writes about the planned and experienced curriculum.^22^ The HPCSA highly regulates the internship of medical doctors and, as such, a planned curriculum leading to certification has been set, approved and monitored. During the COVID-19 pandemic, the interns’ planned learning curriculum was disrupted and replaced by on-the-job learning, a learning informed by the immediate needs of the current medical pandemic situation.

The interns reported learning both soft and hard skills of the medical profession. Soft skills include protocol methodologies, sustained working in a highly compromising environment, developing logical rather than emotional responses to patients and colleagues, and assuming leadership roles in a crisis situation. Hard skills include: ‘knowing the value of IPC [*infection prevention and control*], pandemic preparedness and a first-person perspective of a disaster’ (P12, male, 2nd year). More elaboration on soft skills and hard skills is presented below.

#### Development of interns’ resilience-building skills

Some interns identified deficiencies in managing safety issues, difficult staff, death and dying, whilst others identified clear strategies for coping. In addition, many interns reported emotions of fear, anxiety and being overwhelmed, and they could be self-identifying as cases of burnout. South African doctors, including interns, have documented high background rates of burnout.^9^ Urgent strategies are imperative to mitigate these concerns. The development of resilience has been seen as a means to assist health care workers in similar contexts.^23^ Modifiable factors such as workload, social support, leisure-time activities, access to good mentorship, occupational health and counselling have been shown to influence resilience in clinical areas.^11,24^

#### Consolidate infection prevention and control training and practices

The study found that the interns appreciated and valued IPC protocols put in place as a consequence of COVID-19, such as the *donning and doffing of PPE* (P5). This process has been documented as an important factor in pandemic preparedness.^25^ In addition, the vulnerability felt by the interns in this study about being infected with COVID-19 and being the last ones to have access to PPE can be allayed with IPC protocols being taught, reviewed and practised regularly as part of standard practice.^25^

#### Enhanced core skills training for interns

The interns identified various opportunities for learning as a result of the COVID-19 pandemic. In addition to clinical skills related to high care, intubation and managing hypoxic respiratory distress, the interns also have had the opportunity to enhance other core competencies whilst working collaboratively within teams, for example: ‘organisation, taking leadership roles, compassion within team settings and being a team player’ (P37, male, 1st year). This marks a welcome shift that sees clinicians valuing the view of developing holistic graduate competencies that are important for safe patient management. Whilst undergraduate and postgraduate curricula across SA have used multiple graduate competencies to create frameworks for training and evaluation, the internship programme has not formally embraced this.^26,27^ The COVID-19 pandemic provides a possible and necessary catalyst for an overhaul of assessment and evaluation within internship.

## Limitations

This explorative study is based on the perceptions of the participants. Most of the responses were obtained from one province and thus these perspectives may not reflect a wider country view. Future observational studies with an extended sample might clarify those issues further. This SWOT analysis was conducted only with interns. The analysis should be extended to supervisors, seniors and other hospital staff involved in intern training to get different stakeholder perspectives on the issues found in this study.

## Recommendations

Feasible strategies for present and future pandemics are identified and recommended from the information provided by the interns in this study. These are done specifically to address needs within resource-challenged and disease-burdened contexts. There is a critical need to formally harness existing institutional support systems, including intern curators, occupational health, employment assistance programmes and supervisor–mentors, into developing intern skills training programmes on resilience building. This should include sources of support available and how to avoid negative and enhance positive coping strategies.

The COVID-19 pandemic opportunity must be utilised to formalise curriculum renewal for South African internship in order to ensure that holistic skills are valued and evaluated as part of the internship. The impact of the COVID-19 pandemic on a refocusing on skills outside of specific clinical procedural paradigms has been highlighted by this and other studies. This focus includes improving communication skills, especially related to *breaking bad news* and ‘death and dying’.^28^ A strategy to harness with regard to interns’ self-identified limited knowledge and experience is to leverage their willingness to learn and adjust by creating processes that will facilitate rapid access to information and knowledge. The forced usage of burgeoning online training platforms during the COVID-19 pandemic can serve as a model according to which future intern training can be done, and this has special relevance in resource-constrained contexts. The use of online platforms has the potential to standardise intern training and evaluation across South African hospitals, especially as discrepancies in both have been documented for many years.^8,29^ The compromised learning environment that was created by the need to direct the majority of services to COVID-19 specific care was highlighted as a threat to intern training during the pandemic. Strategies to make up for the decreased scope of learning can thus adopt online learning opportunities to ensure that clinical learning time is more flexible in the internship and that it includes effective feedback. The interns identified weaknesses with feedback during the COVID-19 pandemic. This may reflect the general culture in public-sector hospitals; however, for interns, the need for mentoring and support is especially crucial, and the lack of adequate feedback has negative outcomes.^30^ The creation of formalised feedback systems for interns is overdue, and this needs evaluation once implemented.^31^

## Conclusion

In resource-constrained settings, the COVID-19 pandemic exacerbated existing fault lines within the learning environment of medical interns. Despite these challenges, unique opportunities that can be leveraged to enhance training were identified by the interns themselves. These strategies have the potential to enhance the development of a professional identity amongst emerging medical practitioners in their future roles as community service doctors.
